# Estimates and impact of lymphocyte division parameters from CFSE data using mathematical modelling

**DOI:** 10.1371/journal.pone.0179768

**Published:** 2017-06-16

**Authors:** Pauline Mazzocco, Samuel Bernard, Laurent Pujo-Menjouet

**Affiliations:** 1 Université de Lyon, Université Claude Bernard Lyon 1, CNRS UMR 5558, Laboratoire de Biométrie et Biologie Evolutive, Villeurbanne, France; 2 Université de Lyon, Université Claude Bernard Lyon 1, CNRS UMR 5208, Institut Camille Jordan, Villeurbanne, France; 3 Inria Grenoble Rhône-Alpes Center, Lyon, France; Leids Universitair Medisch Centrum, NETHERLANDS

## Abstract

Carboxyfluorescein diacetate succinimidyl ester (CFSE) labelling has been widely used to track and study cell proliferation. Here we use mathematical modelling to describe the kinetics of immune cell proliferation after an *in vitro* polyclonal stimulation tracked with CFSE. This approach allows us to estimate a set of key parameters, including ones related to cell death and proliferation. We develop a three-phase model that distinguishes a latency phase, accounting for non-divided cell behaviour, a resting phase and the active phase of the division process. Parameter estimates are derived from model results, and numerical simulations are then compared to the dynamics of *in vitro* experiments, with different biological assumptions tested. Our model allows us to compare the dynamics of CD4+ and CD8+ cells, and to highlight their kinetic differences. Finally we perform a sensitivity analysis to quantify the impact of each parameter on proliferation kinetics. Interestingly, we find that parameter sensitivity varies with time and with cell generation. Our approach can help biologists to understand cell proliferation mechanisms and to identify potential pathological division processes.

## 1 Introduction

### 1.1 Biological background and CFSE

Understanding cell proliferation in general, and immune cell dynamics in particular is a great challenge for biologists. Even if tremendous discoveries have been made in the past decades, many mechanisms remain unclear. Our aim here is to focus our attention at the cell population level and more specifically to get the best estimates of the few key parameters able to describe *in vitro* proliferation of immune cells stimulated by an antigen.

To obtain good parameter estimates for cell population dynamics, it is necessary to have time series of experimental data. A good way to get them is to use cell markers. In this work, we study data obtained with carboxyfluorescein diacetate succinimidyl ester (CFSE). It has been shown that CFSE labels resting and proliferating cells regardless of their stage in the division cycle [1, 2]. It binds to intracellular proteins without affecting differentiation or apoptosis during division. Thus experimental data are not biased. Another advantage is that this marker is believed to be equally distributed between the two daughter cells after their mother’s division. Therefore CFSE concentration can be used to count how many divisions a cell has completed. A downside of this method is that its fluorescence can only be detected up to seven or eight divisions due to labelling dilution [3]. Despite this problem, CFSE has been one of the most popular marker because of its ability to track cell proliferation quite efficiently.

### 1.2 Mathematical modelling of cell division

Several mathematical models based on CFSE labelling in cell division have been developed. De Boer and Perelson [4] published a large review of these different models. The simplest one is based on ordinary differential equations (ODE) [5–7]. Although it is simple enough to estimate parameters such as proliferation and death rates [6], this model may not reflect the real biological process of division. Indeed, as division times are implicitly assumed to be exponentially distributed, a cell that has just divided could divide again instantly, which is unrealistic if one accounts for mitosis and DNA replication [6].

An other approach is the cyton model [8, 9]. In this model, times to division and death for each generation of cells are described using independent probability functions. This model is written as a set of integral equations. A general cyton solver (GCytS) [8], coded in Matlab, has been developed for parameter estimation. However, CFSE data are generally not rich enough to correctly estimate the nine parameters in the model.

Hyrien and Zand proposed a branching process model in order to describe CFSE data [10, 11]. This model has been improved by Miao *et al.* [12]. Cells are classified into four subtypes according to the events that occur at the end of a cycle time (death, rest, division or differentiation). This model is a mathematical tool representing cell behaviour and it can predict the average number of cells in different generations as well as the probability to have a certain number of cells in a given generation. Fitting this model to CFSE data provides satisfactory results. However, this type of model is phenomenological, and may fail to explain mechanistic processes.

Finally, some models are based on the Smith-Martin model [13] where the cell cycle is divided into two different phases: a resting phase A with a variable length and a phase B, with a fixed duration, consisting of DNA synthesis, a gap phase G_2_ and a mitotic phase. This model limits proliferation, by introducing a delay between two consecutive divisions. With only four parameters, the duration of phase B, the transition rate from phase A to phase B and the death rates in each phase, the Smith-Martin model is rather simple. However because of identification problems [14], it must be simplified by setting death rates to equal values, reducing the number of parameters to three. Smith-Martin model is able to correctly describe experimental data [6].

Whereas these models describe the evolution of cell numbers in each generation, derived from CFSE histograms, other models, called “label-structured models” deal directly with the fluorescence histogram [15–18], avoiding data pre-processing. Indeed pre-processing can introduce errors, as it is sometimes difficult to assign CFSE intensities to a division number [4]. Moreover, CFSE division in daughter cells could be asymmetric, and these models should overcome this difficulty [19]. Although these models have some similarities to the “age- and division-structured models” that are developed in this study, they deal with a different type of data (fluorescence and not cell numbers) [18].

### 1.3 Objectives

In this study, we aim at estimating parameters related to cell division process, and at quantifying their impact on the dynamics. To do so, we present a model structured in age and division. We base this new model on a study by Bernard *et al.* [20] which was inspired by the Smith-Martin model. We consider here not only phases A and B but also a third phase accounting for the delay between initial stimulation and cell response, to describe non-divided cell behaviour. Because we can derive an explicit solution for the number of cells in each generation, results on existence, uniqueness, non-negativity and behaviour of the solution are straightforward. We then compute parameter estimates and perform a sensitivity analysis to assess the effect of each parameter on the division process.

The paper is organized as follow. We first describe the model presented in [20] and discuss its performance. We then introduce our new model that we believe should describe the data more precisely. In the next section, we present different biological assumptions that we will test in order to get a better experimental fit, along with parameter estimates. We then give an application of parameter estimation to published data. We finally present a sensitivity analysis adapted to our model and discuss our results.

## 2 Mathematical models of cell division

### 2.1 Two-phase model

**Fig 1:**
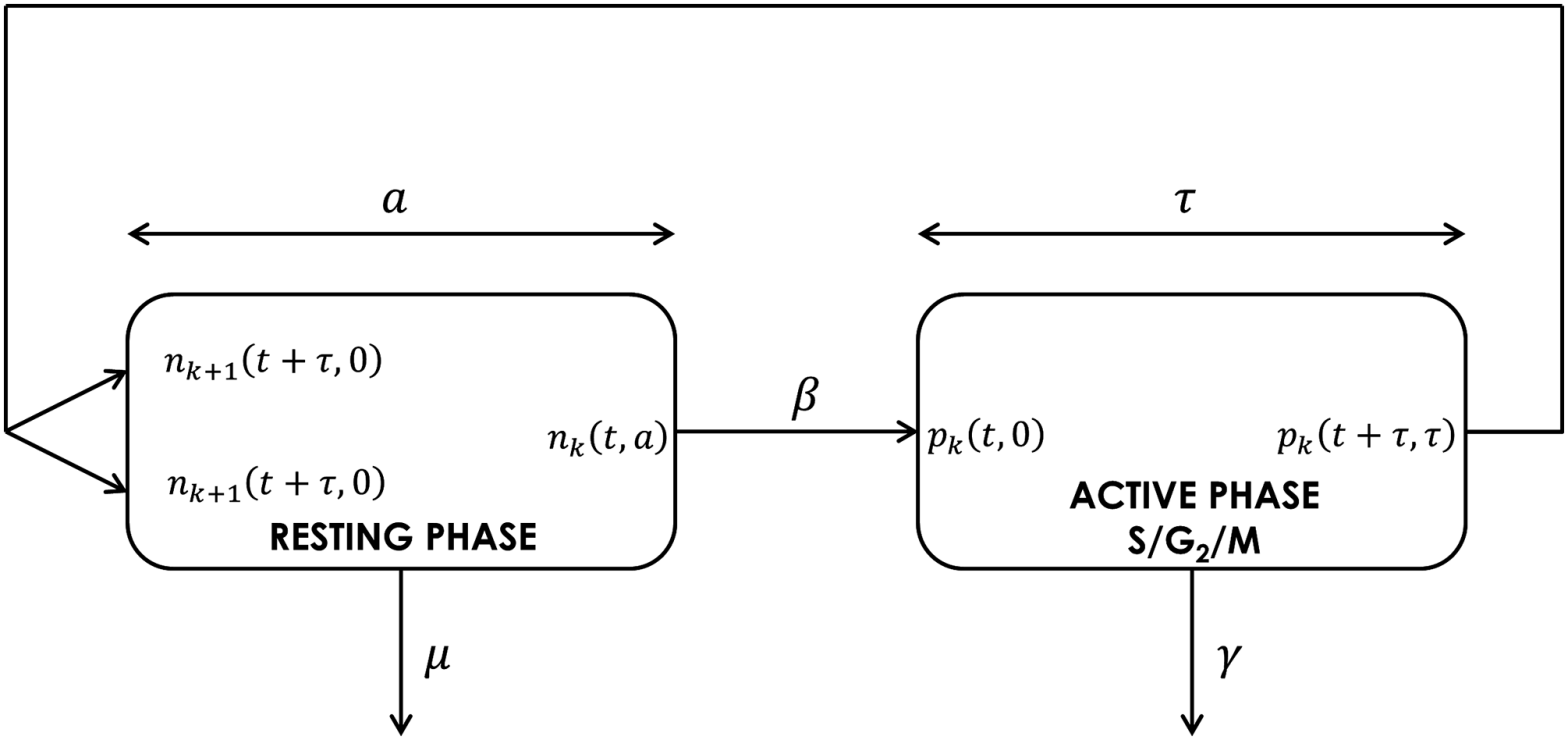
Schematic representation of the cell cycle with a resting phase and an active phase. One can assume that the active phase includes cells which are in S (DNA synthesis), G_2_ (second gap) and M (mitosis). Cells in the active phase can die due to apoptosis at a rate *γ* while *μ* is the loss rate (due to death or differentiation) in the resting phase. *β* is the rate of cell reentry from the resting phase into the active phase, and *τ* is the fixed duration of the active phase.

In 2003, Bernard *et al.* [20] proposed a model based on the Smith-Martin model. Assumptions of this model are the following: the division cycle is divided into two different phases: a resting phase with a random duration, in which cells remain quiescent and an active phase, with a fixed duration. The resting phase may gather both G_0_ and G_1_ phases, while the active phase may consist of DNA synthesis, second gap G_2_ and mitosis phase (see Fig 1). One should note that this distinction is not required to build the model and estimate parameter values but can be helpful to understand the cell division cycle. Bernard *et al.* considered an age-maturity structured model in order to describe CFSE data. This model, which is continuous in time and discrete in maturity, has been called a hybrid model.

The resting phase is probabilistic in the sense that cells can leave this phase at any age, while the active phase is a deterministic one in the sense that all cells surviving this compartment leave it after a fixed time *τ*, called the proliferation time. Using the method of characteristics, this model can be simplified into a system of two delay differential equations. Consequently, the lag time for the division process is not introduced in a phenomenological way, but comes from the mechanistic model and its solutions. The different parameters are assumed to be constant, allowing us to obtain explicit solutions. The hybrid model is described as follows:

$$\begin{array}{c} \left\{ \begin{array}{c} \frac{\partial p{}_{k}\left(t,a\right)}{\partial t}+\frac{\partial p{}_{k}\left(t,a\right)}{\partial a}=-\gamma p{}_{k}\left(t,a\right),\\ \frac{\partial n{}_{k}\left(t,a\right)}{\partial t}+\frac{\partial n{}_{k}\left(t,a\right)}{\partial a}=-\left(\mu +\beta \right)n{}_{k}\left(t,a\right), \end{array}\right. \end{array}$$ (1)

where *p*_*k*_(*t*, *a*) (respectively *n*_*k*_(*t*, *a*)) stands for cell density in the active phase (respectively resting phase) for generation *k* at time *t* ≥ 0 and age *a* ≥ 0.

Boundary conditions used to complete the model are:

$$\begin{array}{c} p{}_{k}\left(t,0\right)=\beta N{}_{k}\left(t\right)=\beta \int_{0}^{\infty }n_{k}\left(t,a\right)da,\;t\geq 0, \end{array}$$ (2)

$$\begin{array}{c} n{}_{k}\left(t,0\right)=2p{}_{k-1}\left(t,\tau \right),\;t\geq 0, \end{array}$$ (3)

where Eq (2) describes the quantity of cells reaching the active phase. This quantity is proportional to the total quantity of cells in the resting phase. Eq (3) accounts for division: 2 daughter cells (of generation *k* + 1) reaching the resting phase come from their mother (of generation *k*) that has just divided at fixed age *τ* in the active phase. It it assumed that initially all cells are undivided and in the resting phase:

$$\begin{array}{c} \left\{ \begin{array}{cc} p_{k}\left(0,a\right)=0, & 0\leq a\leq \tau ,\;\;k\geq 0,\\ n_{0}\left(0,a\right)=R_{0}\delta \left(a\right), & a\geq 0,\\ n_{k}\left(0,a\right)=0, & a\geq 0,\;\;k\geq 1. \end{array}\right. \end{array}$$ (4)

The initial cell number *R*_0_ is assumed to be known. The function *δ*(⋅) is the standard Dirac delta function that represents the fact that all cells have initially an age *a* = 0. In total, this model involves four parameters: *τ*, *β*, *μ* and *γ*. However, in this work, we assume that the two death rates are equal (*μ* = *γ*), reducing the number of parameters to three.

After integrating the two equations in Model (1) with respect to age, and thanks to the method of characteristics, Bernard *et al.* [20] obtained an explicit solution as written below (see [20] for the details). They were able to compute the explicit number of cells in each phase, for each time and generation:

$$\begin{array}{c} n_{k}\left(t,a\right)=R_{0}\frac{{\left(t-a-k\tau \right)}^{k-1}}{(k-1)!}2^{k}{\text{e}}^{-k\tau }\beta^{k}{\text{e}}^{-(\mu +\beta )(t-k\tau )},\;0\leq a\leq t-k\tau , \end{array}$$ (5)

$$\begin{array}{c} p_{k}\left(t,a\right)=R_{0}\frac{{\left(t-a-k\tau \right)}^{k}}{k!}2^{k}{\text{e}}^{-k\gamma \tau }\beta^{k+1}{\text{e}}^{-(\mu +\beta )(t-a-k\tau )}{\text{e}}^{-\gamma a},\;0\leq a\leq t-k\tau , \end{array}$$ (6)

$$\begin{array}{c} N_{k}\left(t\right)=\int_{0}^{\infty }n_{k}\left(t,a\right)da=R_{0}\frac{{\left(t-k\tau \right)}^{k}}{k!}2^{k}{\text{e}}^{-k\gamma \tau }\beta^{k}{\text{e}}^{-(\mu +\beta )(t-k\tau )},\;t\geq k\tau , \end{array}$$ (7)

$$\begin{array}{c} P_{k}\left(t\right)=\int_{0}^{\tau }p_{k}\left(t,a\right)da,\;t\geq 0. \end{array}$$ (8)

However, when comparing numerical simulations with experimental data, Bernard *et al.* [20] concluded that their model was not quite consistent with the observed number of cells in generation 0 and 1. Indeed, although some cells do not divide, the simulations induce an entry in the active phase for almost all the cells. Therefore, simulations suggest that non-divided or one-divided cell populations are cleared after a certain time whereas experimental data shows that some of these cells remain. This discrepancy needed to be accounted for, and this is what is presented in the next section, with a three-phase model.

### 2.2 Three-phase model

**Fig 2:**
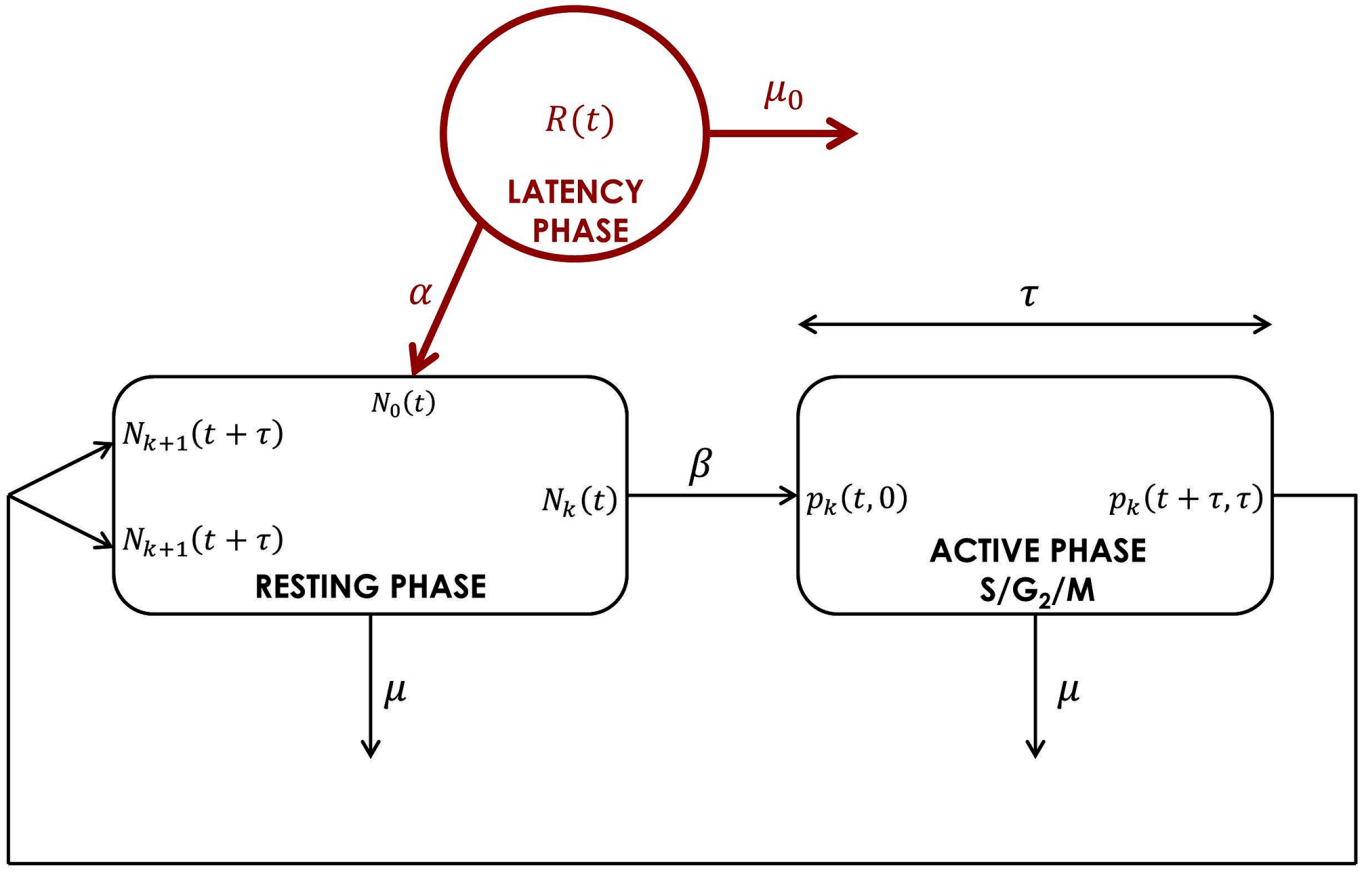
Schematic representation of the cell cycle with a third phase, the latency phase. Although this has no impact on the modelling, one can assume that the resting phase stands for G_0_ and G_1_ phases, while the active phase brings DNA synthesis (S), step G_2_ and mitosis phase together. The rate *α* represents the cell entry from latency phase into the resting phase, and parameter *μ*_0_ is the death rate of cells in the latency phase. All other parameters remain the same as the ones from [20], presented in Section 2.1.

To improve the fitting problem of the two-phase model in the framework of immune cell dynamics, we have developed a new model by adding a third phase (called a latency phase) accounting for the different behaviour of non-divided cells (see Fig 2).

Experimentally, it is observed that once an infectious agent is injected in the body, immune cells need a certain finite time to recognize this agent as a pathogenic one and then they need some specific proteins to be activated [21]. We assume that during this time, cells are in a latency phase, getting ready to leave and proliferate for most of them. Consequently the latency describes the lag time between cell stimulation and cell entry into first division. We assume that all cells are initially located in this latency phase and can leave it at a rate *α* to enter the resting phase of the division cycle. Once cells reach the division cycle they cannot return to the latency phase. Latent cells can die with a rate *μ*_0_. The division cycle is separated into two distinct phases: the resting phase and the active phase at the end of which cells divide.

#### 2.2.1 Model equations

We denote by *R*(*t*) the cell population in the latency phase at time *t*. *N*_*k*_(*t*) denotes the population in the resting phase and *P*_*k*_(*t*) the population in the active phase at time *t* and in the *k*^*th*^ generation. For this extended model the following equations can be derived:

$$\begin{array}{ccc} \frac{dR(t)}{dt}=-\left(\alpha +\mu_{0}\right)R\left(t\right), & & t\geq 0, \end{array}$$ (9)

$$\begin{array}{ccc} \frac{dN_{0}\left(t\right)}{dt}=\alpha R\left(t\right)-\left(\mu +\beta \right)N_{0}\left(t\right), & & t\geq 0, \end{array}$$ (10)

$$\begin{array}{ccc} \frac{dN_{k}\left(t\right)}{dt}=2p_{k-1}\left(t,\tau \right)-\left(\mu +\beta \right)N_{k}\left(t\right), & & t\geq 0,\;\;k\geq 1, \end{array}$$ (11)

$$\begin{array}{ccc} P_{k}\left(t\right)=\beta \int_{0}^{\tau }N_{k}\left(t-a\right){\text{e}}^{-\mu a}da, & & t\geq 0,\;\;k\geq 0, \end{array}$$ (12)

where the term *p*_*k*−1_(*t*, *τ*) in Eq (11) refers to the density of proliferative cells of generation *k* − 1 and age *τ* as described in the two-phase model (Section 2.1).

Eq (9) describes the decrease in cell number in the latency phase due to death and departure to division cycle. Eq (10) describes the behaviour of non-divided cells (generation 0) in the resting phase. Finally, Eq (11) governs the behaviour of cells that have divided more than once (*k*^*th*^ generation) in the resting phase, the term 2*p*_*k*−1_(*t*, *τ*) describing the division of the previous generation. Two different equations for cells in the resting phase are needed since no cell from the latency phase come in generation *k*, when *k* is positive. The number of cells in the active phase is finally computed with Eq (12): cells in the active phase are the ones of the same generation having left resting phase a time *τ* before and having survived the active phase.

To complete the model, a boundary condition is added:

$$\begin{array}{c} p_{k}\left(t,0\right)=\beta N_{k}\left(t\right),\;k\geq 0. \end{array}$$ (13)

This equation represents the fact that cells of generation *k* reaching the active phase are exactly the ones of the same generation, regardless of their age, that come from the resting phase at a rate *β*.

We also assume that all cells are initially in the latency phase:

$$\begin{array}{c} \left\{ \begin{array}{cc} R\left(0\right)=R_{0}>0, & \\ N_{k}\left(0\right)=0, & k\geq 0,\\ p_{k}\left(0,a\right)=0, & 0\leq a\leq \tau \;\;k\geq 0. \end{array}\right. \end{array}$$ (14)

In total, this model involves five parameters. Two parameters have been added compared to the two-phase model, namely the death rate in the latency phase *μ*_0_ and the rate of entry into the resting phase *α*.

Eqs (9) and (10) can easily be solved. Eq (11) is solved as in [20]. Thus we obtain the following solutions for the augmented three-phase latency model:

$$\begin{array}{c} R\left(t\right)=R\left(0\right){\text{e}}^{-(\alpha +\mu_{0})t},\;t\geq 0, \end{array}$$ (15)

$$\begin{array}{c} N_{0}\left(t\right)=R\left(0\right)\frac{\alpha }{\left(A-B\right)}\left({\text{e}}^{-(\alpha +\mu_{0})t}-{\text{e}}^{-(\mu +\beta )t}\right),\;t\geq 0, \end{array}$$ (16)

$$\begin{array}{c} N_{k}\left(t\right)=R\left(0\right)\frac{{\left(2\beta {\text{e}}^{-\mu \tau }\right)}^{k}\alpha }{{\left(A-B\right)}^{k+1}}{\text{e}}^{-A(t-k\tau )}\sum_{l=k+1}^{+\infty }\frac{{\left[(A-B)(t-k\tau )\right]}^{l}}{l!},\;t\geq k\tau ,\;\;k\geq 1, \end{array}$$ (17)

where *A* = *μ* + *β* and *B* = *α* + *μ*_0_, and *P*_*k*_(*t*) is computed using Eq (12). Note that the computation of this integral is tedious. This is the reason why we keep this form for the solution and compute it numerically.

#### 2.2.2 Model analysis

**Proposition 1** *Existence and uniqueness of solutions*

*The system of Eqs* (9)–(11) *has one unique solution, given by Eqs* (15)–(17).

*Moreover, let us assume that* (*μ* + *β*) *is greater than* (*α* + *μ*_0_). *Under this condition, and for non-negative initial conditions, solutions are non-negative*.

*proof.* We are able to compute the solutions of our system of equations, which prove their existence and uniqueness. However, existence of Eq (17) may be an issue due to the sum $\sum_{l=k+1}^{+\infty }\frac{{\left[(A-B)(t-k\tau )\right]}^{l}}{l!}$. This sum can be expanded as:

$$\begin{array}{c} \sum_{l=k+1}^{+\infty }\frac{{\left[(A-B)(t-k\tau )\right]}^{l}}{l!}=\exp(\left(A-B)(t-k\tau \right))-\sum_{l=0}^{k}\frac{{\left[(A-B)(t-k\tau )\right]}^{l}}{l!}, \end{array}$$ (18)

which shows that it does exist.

Non-negativity of solutions is straightforward, given their expressions (Eqs (15)–(17)).

**Proposition 2** *Steady state*

*The model admits a unique steady state* $\bar{R}=0$, $\bar{N_{k}}=0,k\geq 0$, $\bar{P_{k}}=0,k\geq 0$. *Moreover, for all* *k* ≥ 0 *N*_*k*_ *and* *P*_*k*_ *tend to 0 as* *t* *tends to infinity*.

*proof.* This result is easily obtained from the explicit formulae of solutions.

This steady state implies that generation *k* eventually empties, for all *k*, but the cell population continues to grow, as cells can divide indefinitely. However, if we assume that cells cannot divide more than *K*_*max*_ > 0 times, all cells will eventually die due to the loss term. Indeed, an extra equation would be needed in the model to describe the behaviour of cells in this maximal generation, with parameter *β* set to zero. One can easily see that the steady state remains unchanged, with $\bar{N_{K_{max}}}=\bar{P_{K_{max}}}=0$.

## 3 Model selection and parameter estimates

### 3.1 Testing biological assumptions to improve our model

With the enhanced three-phase model introduced in Section 2.2, we can compute the number of cells in each generation at different times and compare model predictions to existing data. Several scenarios can also be tested in order to get a better data fit.

First it seems biologically reasonable to study the model without cell death during the latency phase. From a biological point of view, cells can die because of a damaged DNA detected at the G_2_ phase checkpoint, so there is no reason for cells to die during the latency phase while they are only quiescent. Therefore, it appears legitimate to test our model with parameter *μ*_0_ equal to 0.

Secondly, in previous papers, it was claimed that undivided cells do not die at the same rate as the other cells [12, 22]. A cell going through many divisions could have more risk of dying because of a damaged DNA due to replication than a cell which has divided only once or twice. Thus, it seems also realistic to test our model with *μ* linearly dependent on the number of divisions as proposed in [22].

Each hypothesis is tested separately and together:

Scenario 1, *μ*_0_ = 0: no cell death during the latency phase is considered. The cells can only die once they have entered the division cycle. This model involves four free parameters.
Scenario 2, *μ*_0_ ≠ 0: cells can die during the latency phase to account for the loss that might be observed in the first day of the experiment. This model involves five free parameters.
Scenario 3, *μ*_0_ = 0 and *μ* = *f*(*k*): as found in [22] the death rate might depend on the cell generation in a linear fashion: *μ* = max(*μ*_*k*,0_ + *k* × *μ*_*k*_; 0). This guarantees *μ* is non-negative, by taking *μ* = 0 if *μ*_*k*,0_ + *k* × *μ*_*k*_ is negative. This model involves five free parameters.
Scenario 4, *μ*_0_ ≠ 0 and *μ* = *f*(*k*): a combination of the two different losses explained above in Scenario 2 and Scenario 3. Cells can die during the latency phase and the death rate during the division process depends of the number of divisions through the same relation as before. This model involves six free parameters.

The different scenarios are compared using the standard selection criteria AICc [23, 24], which can be written as $AICc=2k-2\text{ln}\left(L\right)+\frac{2k(k+1)}{n-k-1}$, where *k* is the number of parameters, *n* is the number of observations and *L* represents the likelihood of the model. AICc can also be written as follows:

$$\begin{array}{c} AICc=n\text{ln}\left(\frac{LSS}{n}\right)+2k+\frac{2k(k+1)}{n-k-1}, \end{array}$$ (19)

where *LSS* is the sum of the squares of residuals (differences between model prediction and experimental data), obtained from least-square model fitting. *LSS* can simply be written as *LSS* = ∑(*y* − *f*)^2^, where *y* represents the observations and *f* represents the simulations derived from the model. Eq (19) is a simplified equation for AICc, in which we assume a Gaussian error model. This assumption allows us to easily compare performances of our different hypotheses, in term of data fitting, without actually building a more complex error model. We compute Δ*AICc* = *AICc*_*i*_ − *AICc*_*base*_ where *AICc*_*i*_ is the value of AICc for three-phase model and scenario *i* and *AICc*_*base*_ is the value of AICc for the two-phase model. Δ*AICc* measures the quality of a model in terms of fitting but also of model complexity, expressed as the number of parameters. This criteria rewards the better fit to experimental data and, at the same time, penalizes over-parametrized models. A negative value of Δ*AICc* corresponds to an improvement of the model, in the sense of AICc. Indeed, a model is considered better than an other one, if its value of AICc is lower.

We also use a likelihood-ratio test based on least square sum [25], to determine whether the addition of parameters is significant or not. Although AICc can be used to compare any models, this statistical test is used to compare nested models only. Two models are nested if the first one can be transformed in the second model through constraints on the parameters of the first model. The different scenarios for the three-phase model are nested models, and can therefore be compared with a likelihood-ratio test. The statistical test actually addresses the question whether the “true” parameters can be found among the subset with constraints. The test statistic writes $U_{n}=n\frac{LSS_{2}-LSS_{1}}{LSS_{1}}$, where *n* is the number of observations, *LSS*_1_ is the least square sum of the complete model and *LSS*_2_ is the least square sum of the model with constraints (*ie* the model with fewer parameters). Under the null hypothesis (“true” parameters can be found in the subset with constraints) this statistic follows a *χ*^2^ distribution. We reject the null hypothesis if the test statistic occurs with a probability *p* < 0.05.

### 3.2 Parameter estimates

Our goal here is to obtain parameter estimates with which we can reproduce the observed cell population dynamics for a given time series in a consistent way. To reach this objective, we first derive parameter estimates for a given time of the experiment. We claim that a parameter set estimated using only the last time point provides an accurate initial guess to start an estimation algorithm. In this work, we choose to use the Levenberg-Marquardt nonlinear least squares algorithm [26, 27] implemented in the software MATLAB^®^ (Release 2014b, The MathWorks, Inc, Natick, MA, US). In the next two sections, we provide formulae to estimate parameters of the two-phase and the three-phase models. Similar work has been performed in [6] for an ODE model.

#### 3.2.1 Estimates for the two-phase model (presented in Section 2.1)

First, we estimate parameters of the two-phase model. We assume that the rate of cell death is the same during the whole division cycle (*γ* = *μ* in Eqs (7) and (8)). Indeed, given the experimental data, the distinction between the two death rates is quite challenging, and causes identifiability issues. Likewise, setting *μ* or *γ* equal to zero prevents us from obtaining explicit formulae for parameter estimates.

We first derive an estimate for the parameter *τ*, which represents the duration of the proliferative phase. All our equations are valid if *t* is greater than or equal to *kτ*, with *t*, *k* and *τ* non-negative. The largest number of division that can be observed by time *t* is $k_{max}\left(t\right)=E\left(\frac{t}{\tau }\right)$, where *E* is the floor function, *ie* the integer part of the argument. Therefore, we have:

$$\begin{array}{c} \tau_{inf}=\frac{t}{k_{max}^{obs}+1}<\tau \leq \frac{t}{k_{max}^{obs}}=\tau_{sup}, \end{array}$$ (20)

where $k_{max}^{obs}$ is the number of divisions actually observed during the experiment. We choose, as a first estimate for the parameter *τ*,

$$\begin{array}{c} {\hat{\tau }}^{1}=\frac{\tau_{inf}+\tau_{sup}}{2}. \end{array}$$ (21)

We then focus on estimating parameter *β*, the entry rate from the resting phase to the active phase. The theoretical mean number of cell divisions will be compared to the observed one. Denote by *X* the random variable representing the number of divisions a cell has been through. We have the following probability distribution:

$$\begin{array}{c} P\left(X=k\right)=p_{k}\left(t\right)=\frac{T_{k}\left(t\right)}{T(t)},\;\text{for}\;k\geq 0, \end{array}$$ (22)

where *T*_*k*_(*t*) is the total number of cells of generation *k* at time *t*, and *T*(*t*) is the total number of cells, regardless of the number of divisions, at time *t*. We first give expressions for *N*(*t*), *P*_*k*_(*t*) and *P*(*t*). From Eq (7), we obtain:

$$\begin{array}{ccc} N(t) & = & \sum_{k=0}^{k_{max}}N_{k}\left(t\right),\\ & = & {\text{e}}^{-\mu t}\sum_{k=0}^{k_{max}}\frac{{\left(t-k\tau \right)}^{k}}{k!}2^{k}\beta^{k}{\text{e}}^{-\beta (t-k\tau )},\\ N(t) & = & {\text{e}}^{-\mu t}S_{N}\left(t,\tau ,\beta \right), \end{array}$$ (23)

where

$$\begin{array}{c} S_{N}\left(t,\tau ,\beta \right)=\sum_{k=0}^{k_{max}}\frac{{\left(t-k\tau \right)}^{k}}{k!}2^{k}\beta^{k}{\text{e}}^{-\beta (t-k\tau )}. \end{array}$$ (24)

From Eq (8), we obtain:

$$\begin{array}{ccc} P_{k}\left(t\right) & = & \int_{0}^{\tau }p_{k}\left(t,a\right)da,\\ & = & \int_{0}^{\tau }\left(\frac{{\left(t-a-k\tau \right)}^{k}}{k!}2^{k}{\text{e}}^{-k\tau \mu }\beta^{k+1}{\text{e}}^{-(\mu +\beta )(t-a-k\tau )}{\text{e}}^{-\mu a}\right)da,\\ P_{k}\left(t\right) & = & {\text{e}}^{-\mu t}\int_{0}^{\tau }\left(\frac{{\left(t-a-k\tau \right)}^{k}}{k!}2^{k}\beta^{k+1}{\text{e}}^{-\beta (t-a-k\tau )}\right)da. \end{array}$$ (25)

Therefore the total number of cells in the proliferative phase at time *t* ≥ 0 is:

$$\begin{array}{ccc} P(t) & = & \sum_{k=0}^{k_{max}}P_{k}\left(t\right),\\ & = & {\text{e}}^{-\mu t}\sum_{k=0}^{k_{max}}\int_{0}^{\tau }\left(\frac{{\left(t-a-k\tau \right)}^{k}}{k!}2^{k}\beta^{k+1}{\text{e}}^{-\beta (t-a-k\tau )}\right)da,\\ P(t) & = & {\text{e}}^{-\mu t}S_{P}\left(t,\tau ,\beta \right), \end{array}$$ (26)

where

$$\begin{array}{c} S_{P}\left(t,\tau ,\beta \right)=\sum_{k=0}^{k_{max}}\int_{0}^{\tau }\left(\frac{{\left(t-a-k\tau \right)}^{k}}{k!}2^{k}\beta^{k+1}{\text{e}}^{-\beta (t-a-k\tau )}\right)da. \end{array}$$ (27)

We can now easily compute *T*_*k*_(*t*) = *N*_*k*_(*t*)+*P*_*k*_(*t*) and *T*(*t*) = *N*(*t*)+*P*(*t*):

$$\begin{array}{ccc} T_{k}\left(t\right) & = & {\text{e}}^{-\mu t}F_{k}\left(t,\tau ,\beta \right), \end{array}$$ (28)

$$\begin{array}{c} T\left(t\right)={\text{e}}^{-\mu t}\left(S_{N}\left(t,\tau ,\beta \right)+S_{P}\left(t,\tau ,\beta \right)\right), \end{array}$$ (29)

where *S*_*N*_(*t*, *τ*, *β*) and *S*_*P*_(*t*, *τ*, *β*) are given in Eqs (24) and (27), and

$$\begin{array}{c} F_{k}\left(t,\tau ,\beta \right)=\frac{{\left(t-k\tau \right)}^{k}}{k!}2^{k}\beta^{k}{\text{e}}^{-\beta (t-k\tau )}+\int_{0}^{\tau }\left(\frac{{\left(t-a-k\tau \right)}^{k}}{k!}2^{k}\beta^{k+1}{\text{e}}^{-\beta (t-a-k\tau )}\right)da. \end{array}$$ (30)

The probability distribution (Eq (22)) is thus independent of the parameter *μ* and is given by

$$\begin{array}{c} p_{k}\left(t\right)=\{\begin{array}{cc} \frac{F_{k}\left(t,\tau ,\beta \right)}{S_{N}\left(t,\tau ,\beta \right)+S_{P}\left(t,\tau ,\beta \right)}, & 0\leq k\leq k_{max},\\ 0, & k>k_{max}. \end{array} \end{array}$$ (31)

The parameter *β* can then be estimated by comparing the theoretical mean number of divisions *m*_*theo*_(*t*, *τ*, *β*), computed with probability distribution Eq (31), to the one from experimental data *m*_*obs*_:

$$\begin{array}{c} {\hat{\beta }}^{1}=\underset{\beta }{\mathrm{argmin}}\left({\left(m_{theo}\left(t,{\hat{\tau }}^{1},\beta \right)-m_{exp}\right)}^{2}\right). \end{array}$$ (32)

We are now able to estimate the parameter *μ*, standing for the rate of death, using Eq (29). We obtain:

$$\begin{array}{c} {\hat{\mu }}^{1}=-\frac{1}{t}\ln\left(\frac{T(t)}{S_{N}\left(t,{\hat{\tau }}^{1},{\hat{\beta }}^{1}\right)+S_{P}\left(t,{\hat{\tau }}^{1},{\hat{\beta }}^{1}\right)}\right). \end{array}$$ (33)

#### 3.2.2 Estimates for the three-phase model (presented in Section 2.2)

Now, parameters from the three-phase model are estimated using model results (Eqs (12) and (15)–(17)) and experimental observations. We first present the estimates for Scenario 1, assuming that *μ*_0_ is equal to 0 and that *μ* is independent of the number of divisions. Parameter *α*, standing for the rate of entry from the latency phase to the division process, can be estimated by comparing the theoretical number of undivided cells *T*_0_(*t*) to the experimental one:

$$\begin{array}{ccc} T_{0}\left(t\right) & = & R\left(0\right)[{\text{e}}^{-\alpha t}+\frac{\alpha }{\mu +\beta -\alpha }{\text{e}}^{-\alpha t}+\frac{\beta \alpha }{\left(\mu +\beta -\alpha )(\alpha -\mu \right)}{\text{e}}^{-\alpha t}\left({\text{e}}^{(\alpha -\mu )\tau }-1\right)]\\ & & -\frac{R(0)\alpha }{\mu +\beta -\alpha }{\text{e}}^{-(\mu +\beta )t}{\text{e}}^{\beta \tau }. \end{array}$$ (34)

Then,

$$\begin{array}{c} {\hat{\alpha }}^{1}=\underset{\alpha }{\mathrm{argmin}}\left({\left(T_{0}\left(t\right)-T_{0}^{obs}\right)}^{2}\right), \end{array}$$ (35)

where *T*_0_(*t*) is computed with the estimates ${\hat{\tau }}^{1}$, ${\hat{\beta }}^{1}$ and ${\hat{\mu }}^{1}$, obtained with the two-phase model (Eqs (21), (32) and (33)). Because these values were obtained with the previous model, they should now be updated, using the results from the three-phase model. Parameters *τ* and *β* are estimated using the theoretical mean number of divisions, as they are both directly linked to the observed number of divisions. Because the three-phase model is more complex, we are not able to obtain formulae independent of one or another parameter. The estimate for the parameter *μ* is then obtained by comparing the theoretical total number of cells to the experimental one. Finally, parameter *α* is estimated a second time with the new values for *τ*, *β* and *μ*. For the other scenarios (2, 3 and 4), given in Section 3.1, the same method of estimation is used. For scenarios 2 and 4, *α* and *μ*_0_ are estimated together, using the number of non-divided cells given in Eq (34). For scenarios 3 and 4, *μ*_*k*,0_ and *μ*_*k*_ are estimated using Eq (29), assuming that *μ* = max(*μ*_*k*,0_ + *k* × *μ*_*k*_; 0).

These estimates are computed using the last time observation. They enable us to adequately reproduce experimental data for all times. Therefore, from our point of view, they constitute a rather satisfactory initial set of parameters to start a Levenberg-Marquardt algorithm.

## 4 Application to immune cells

### 4.1 Data

We use previously published data [28] as a benchmark to estimate model parameters. This dataset was also used in [12] to compare different models. We apply our model to the same dataset, ensuring a consistent comparison with [12].

Data consists of heparinized blood samples collected from anonymous healthy human donors. CD4+ and CD8+ T cells were isolated from these samples and labelled with CFSE. The evolution of these cells was observed after stimulation with a polyclonal agent, PHA, which is a T-cell activator. The number of cells in each generation (from 0 to 6) was measured at 0, 24, 48, 72, 96, 108 and 120 hours using CFSE profiles.

### 4.2 Analysis of CD4+ cells

We first study CD4+ cell behaviour, and estimate parameter values for the different models and hypotheses presented above. To compare non-nested models, we use the AICc value (given in Section 3.1), and more precisely the Δ*AICc* value. Results are reported in Table 1.

**Table 1:** Values of selection criteria for the different models tested, with CD4+ cell data. The best result is obtained with the three-phase model and scenario 4 (shaded area).

Models	AICc	Δ*AICc*
2-phase model	986	0
3-phase model, scenario 1 (*μ*_0_ = 0)	981	−5
3-phase model, scenario 2 (*μ*_0_ ≠ 0)	918	−68
3-phase model, scenario 3 (*μ*_0_ = 0 and *μ* = *f*(*k*))	942	−44
**3-phase model, scenario 4** (*μ*_0_ ≠ 0 **and** *μ* = *f*(*k*))	**909**	**-77**

According to Δ*AICc* values, we notice that the three-phase model performs better than the two-phase model, regardless of the hypothesis made on *μ*_0_ or *μ*_*k*_. The lowest value is obtained for the three-phase model and scenario 4, assuming *μ*_0_ ≠ 0 and *μ*_*k*_ dependent on the number of divisions. We next compare nested models, by using the likelihood-ratio test presented in Section 3.1. Results are reported in Table 2, with p-values of the test.

**Table 2:** p-values of the statistical test used to compare nested model, with CD4+ cell data. We consider that the difference between two models is significant if the p-value is less than 0.05. Scenarios 2 and 3 are not nested.

3-phase model	S2, *μ*_0_ ≠ 0	S3, *μ*_0_ = 0, *μ* = *f*(*k*)	S4, *μ*_0_ ≠ 0, *μ* = *f*(*k*)
S1, *μ*_0_ = 0	*p* < 0.001	*p* < 0.001	*p* < 0.001
S2, *μ*_0_ ≠ 0	–	–	*p* < 0.001
S3, *μ*_0_ = 0, *μ* = *f*(*k*)	–	–	*p* < 0.001

As we can see in Table 2, all nested models are significantly different. The most complex model (three-phase model with scenario 4) provides a significantly better fit, compared to the other models. The best results are obtained with the set of parameters reported in Table 3, estimated using the method described in Section 3.2.2.

**Table 3:** Parameter estimates for the three-phase model with scenario 4, to describe CD4+ cell behaviour.

Parameters	Units	Estimates	95%-Confidence interval
*α*	d^−1^	0.1921	(0.1375, 0.2467)
*β*	d^−1^	1.4777	(1.0449, 1.9105)
*τ*	d	0.5074	(0.3990, 0.6157)
*μ*_0_	d^−1^	1.0602	(0.8413, 1.2790)
*μ*_*k*,0_	d^−1^	−0.3214	(-0.5104, -0.1324)
*μ*_*k*_	d^−1^	0.1120	(0.0591, 0.1649)

**Fig 3:**
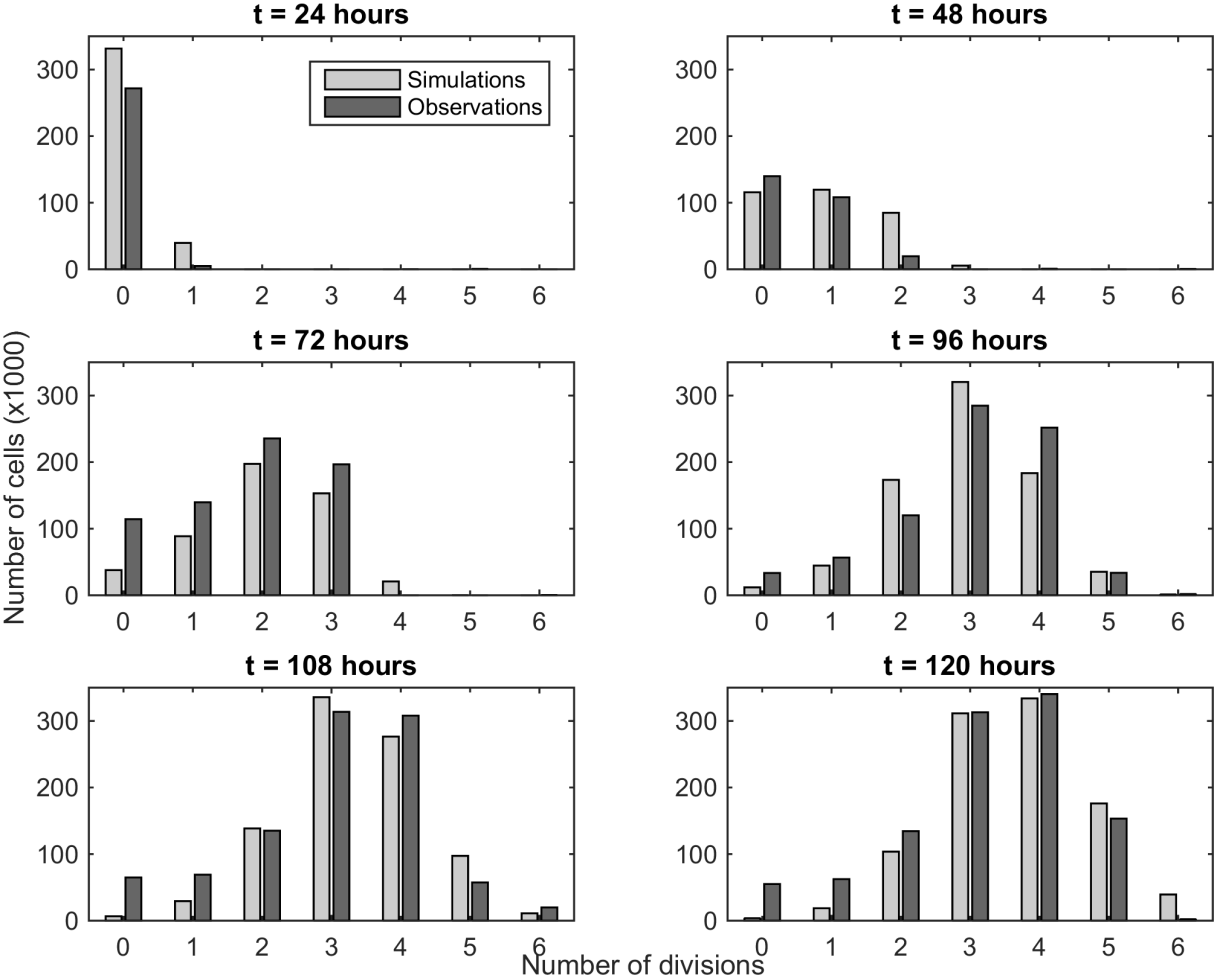
Comparison between numerical simulations and observations of CD4+ cells. Numerical simulations are represented in light grey while experimental data are in dark grey. Cell death during the latency phase is allowed and the rate of death depends on the number of divisions. These results are obtained with the parameter values reported in Table 3.

As explained in Section 3.1, the value of *μ* = *μ*_*k*,0_ + *kμ*_*k*_ is set to 0 if *μ*_*k*,0_ + *kμ*_*k*_ is negative, which is the case here for *k* = 0, 1, 2. Therefore only cells that have divided at least three times can die during the division process. Since *μ*_*k*_ is greater than 0, the probability that cells die when they divide increases with the number of divisions. Moreover, we keep the hypothesis that *μ*_0_ is not equal to 0, whereas it seems that cells in latency phase have no reason to die. However, a strong loss is observed at the beginning of the experimental study, due to the *in vitro* experiment. According to the value of *β* and *τ*, CD4+ cells spend 16 hours in resting phase, and 12 hours in the active phase of the division process, meaning that they divide in average every 28 hours (once per day). Fig 3 displays the results obtained with the three-phase model and scenario 4 (light grey), as well as the observed data (dark grey), for each generation and for all times of observation. With this model, we are able to correctly reproduce CD4+ cell behaviour during the division process, and the latency phase allows for a delay of the entry of cells into division.

### 4.3 Analysis of CD8+ cells

We now study the behaviour of CD8+ cells, and estimate the different parameters for the two models and the different scenarios. We compute the values of the selection criteria, AICc and Δ*AICc*, and report them in Table 4.

**Table 4:** Values of selection criteria for the different models tested with CD8+ cell data. The best result is obtained with the three-phase model and scenario 4 (shaded area).

Models	AICc	Δ*AICc*
2-phase model	1053	0
3-phase model, scenario 1 (*μ*_0_ = 0)	1025	−28
3-phase model, scenario 2 (*μ*_0_ ≠ 0)	1026	−27
3-phase model, scenario 3 (*μ*_0_ = 0 and *μ* = *f*(*k*))	1026	−27
**3-phase model, scenario 4** (*μ*_0_ ≠ 0 **and** *μ* = *f*(*k*))	**1003**	**-50**

Once again, the three-phase model performs better than the two-phase model, although differences in AICc values are less important than for CD4+ cells. We then compare nested models, to determine whether the differences are significant. p-values are reported in Table 5.

**Table 5:** p-values of the statistical test used to compare nested model, with CD8+ cell data. We consider that the difference between two models is significant if the p-value is less than 0.05.

3-phase model	S2, *μ*_0_ ≠ 0	S3, *μ*_0_ = 0, *μ* = *f*(*k*)	S4, *μ*_0_ ≠ 0, *μ* = *f*(*k*)
S1, *μ*_0_ = 0	*p* = 0.19	*p* = 0.27	*p* < 0.001
S2, *μ*_0_ ≠ 0	–	–	*p* < 0.001
S3, *μ*_0_ = 0, *μ* = *f*(*k*)	–	–	*p* < 0.001

Although scenario 2 and scenario 3 have lower AICc values than scenario 1, we observe that the difference is not significant, meaning that we cannot reject the hypothesis that the “true” parameters can be found in the subset with the constraint described by the scenario 1. However, estimating *μ*_0_ and a death rate dependent on the number of divisions gives a significantly better fit. Consequently, the three-phase model with death in the latency phase, and a death rate depending on the number of division best describes CD8+ cell behaviour. The best results are obtained with the set of parameters reported in Table 6.

**Table 6:** Parameter estimates for the three-phase model with scenario 4, to describe CD8+ cell behaviour.

Parameters	Units	Estimates	95%-Confidence interval
*α*	d^−1^	0.1745	(0.1212, 0.2279)
*β*	d^−1^	13.4731	(-0.1856, 27.1319)
*τ*	d	0.6057	(0.5348, 0.6766)
*μ*_0_	d^−1^	0.4524	(0.3030, 0.6017)
*μ*_*k*,0_	d^−1^	−0.4442	(-0.6621, -0.2264)
*μ*_*k*_	d^−1^	0.1287	(0.0927, 0.1647)

We notice that because of the values of parameters *μ*_*k*,0_ and *μ*_*k*_, the rate of death during the division process increases with the number of divisions, but is equal to 0 for *k* = 0, 1, 2, 3. For the same reason as for CD4+ cells, we add a loss term during the latency phase, which accounts for the loss observed experimentally. From the values of *β* and *τ*, CD8+ cells spend on average 2 hours in the resting phase and 14 hours in the active phase. Thus, they divide every 16 hours in average, more than 1.5 times faster than CD4+ cells.

**Fig 4:**
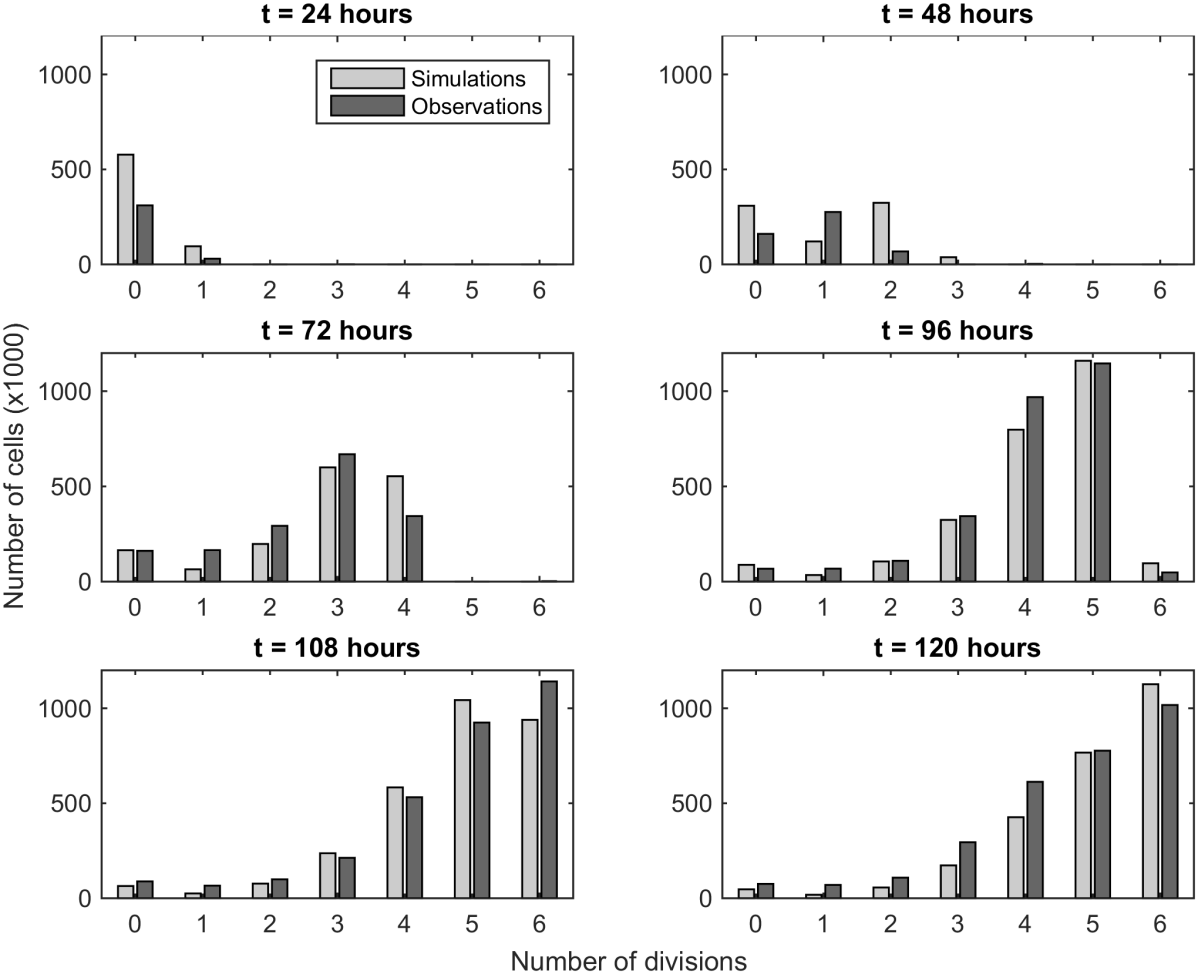
Comparison between numerical simulations and observations of CD8+ cells. Numerical simulations are represented in light grey while experimental data are in dark grey. Cell death during the latency phase is allowed and the rate of death depends on the number of divisions. This result is obtained with the parameter values reported in Table 6.

Fig 4 displays the result obtained with the three-phase model and scenario 4 (light grey), as well as the observed data (dark grey), for each generation and for all times of observation, for CD8+ cells. Parameter estimates (Table 6) allow us to correctly reproduce the behaviour of these cells for each generation and time point.

### 4.4 Comparison between CD4+ and CD8+ cell division kinetics

For both CD4+ and CD8+ cells, we showed that the three-phase model performs better than the two-phase model, the latency phase preventing a too rapid first division. This allows us to compare kinetic behaviours of the two types of cells (see Table 7).

**Table 7:** Comparison of CD4+ cells and CD8+ cells behaviour.

	CD4+ cells	CD8+ cells
Duration of active phase	12 hours	14 hours
Duration of resting phase	16 hours	2 hours
Duration of a division	28 hours	16 hours
Cell death during latency phase	Yes	Yes
Death rate *μ* = *f*(*k*)>0	For *k* ≥ 3	For *k* ≥ 4

We find that CD8+ cells have a faster kinetic than CD4+ cells. Indeed, after the first division, CD8+ cells divide every 16 hours on average, whereas we observe one division every 28 hours on average for CD4+ cells. This difference in kinetics is visible in the experimental data sets, as we notice that at the end of the experiment many CD8+ cells have divided at least 6 times. According to our results, this is mainly due to the time spent in the resting phase: while CD8+ cells stay 2 hours in average in this phase, CD4+ cells leave the resting phase after 16 hours. Moreover, as the resting phase duration is assumed to be exponentially distributed, the parameter *β* also represents the variability of this distribution. Thus, the difference in *β* values implies that CD4+ cells show more variability in division kinetics than CD8+ cells. Then both cell populations spend similar amount of time in active phase. For comparison, Miao *et al.* [12] reported that the average time to division was 19.3 hours for CD4+ cells and 15.1 hours for CD8+ cells. They thus found that CD4+ and CD8+ cells have similar division kinetics, except for the first division, CD4+ cells being slower than CD8+ cells. In our study, we find that both cells have a similar time to first division, but CD8+ cells then re-enter into the active phase of division more rapidly. This is in agreement with the results reported in [29] and [30], concluding that CD8+ and CD4+ cells have a similar response to stimulation, except that CD8+ cells have faster kinetics. The parameter of cell loss during the latency phase remains difficult to interpret, as it accounts for the loss due to the experiment and not only due to the division process. The same observation can be done for the parameter *α*, representing the entry from the latency phase into the resting phase. In our results, this parameter has a small value, which could mean that the time to first division is very long. However, the value of *α* is correlated to the value of *μ*_0_: if many cells die when the study starts, only a few can enter into the division process. This difficulty may be overcome by estimating the number of cells lost because of the experiment, so that we only study the surviving cells that will effectively divide.

### 4.5 Comparison with the branching process models and the Cyton model

We then compare our results to the ones published by Miao *et al.* in [12], where the same dataset is used. It appears that Miao *et al.* use data in log_10_ scale to estimate model parameters.

In a first step, we recomputed the sum of the squares of residuals (*LSS*) and the AICc value, using data and simulations in log_10_ scale. However, our simulation results had been obtained with a parameter set based on raw data. Consequently, this parameter set may not be the optimal one to fit data in log_10_ scale. Therefore, in a second step, we estimated new model parameters using data in log_10_ scale. Values of *LSS* and AICc for the different models are presented in Table 8.

**Table 8:** Comparison with results from [12] (branching process models and Cyton model). *LSS* and AICc values for the three-phase model and scenario 4 are computed using parameters reported in Tables 3 and 6 (with raw data) and with re-estimated parameters (with log_10_ data).

Data	Model	*LSS*	AICc
CD4+ cells	Branching process model 1 [12]	59.0	45.1
	Branching process 2 [12]	59.9	38.8
	Cyton model [12]	60.2	39.1
	3-phase model and scenario 4, with raw data	86.7	42.0
	3-phase model and scenario 4 with log_10_ scale	67.3	29.6
CD8+ cells	Branching process model 1 [12]	42.1	28.6
	Branching process 2 [12]	42.0	21.5
	Cyton model [12]	49.4	29.4
	3-phase model and scenario 4, with raw data	64.3	27.3
	3-phase model and scenario 4 with log_10_ scale	54.4	19.1

We observe that for both CD4+ and CD8+ cells, our results are similar with the ones reported in [12], in terms of AICc values. Specifically, when parameters are estimated with raw data, the value of *LSS* is greater for both CD4+ and CD8+ cells compared with *LSS* values for branching process models and Cyton model. However, the value of AICc is lower than the one for the branching process model 1 for CD4+ cells, and lower than the ones for the branching process model 1 and the Cyton model for CD8+ cells. When parameters are re-estimated using data in log_10_ scale (as done in [12]), we obtain higher values of *LSS* but, because the current model has fewer parameters, the AICc values are lower than the three other models for both cell types. Consequently, our model performs better than the branching process models and the Cyton model in terms of AICc values.

## 5 Sensitivity analysis

We perform a global sensitivity analysis to assess the impact of model parameters on model output. In our case, we can study the effect of each parameter on the total number of cells, for each time and each generation, through first order Sobol’ indices [31] given by the following expression:

$$\begin{array}{c} S_{i}=\frac{V(E\left[Y|X_{i}\right])}{V(Y)},\;i=1,\cdots N, \end{array}$$ (36)

where *Y* is the model output (number of cells in a given generation and for a given time in our case), *X*_*i*_ is a model parameter, *V*(*Y*) represents the total variance of *Y*, **E**[*Y*|*X*_*i*_] is the conditional mean of *Y* given *X*_*i*_, and *N* is the number of model parameters. Note that Sobol’ indices are always between 0 and 1. *S*_*i*_ measures the part of *Y* variance that is explained by parameter *X*_*i*_. In other terms, first order Sobol’ indices determine how much the model output varies when a parameter value varies. A parameter associated with a Sobol’ index close to 1 has a large impact on *Y* variability, meaning that the model output is very sensitive to change in this parameter. A sensitivity analysis can be used in models with a large number of parameters to determine which ones contribute most to the output. In this work, we propose to apply a sensitivity analysis to highlight the impact of each parameter during the division process. We limit our study to first order Sobol’ indices. Note that the impact of the interaction of several parameters on model output can be also be assessed through Sobol’ indices, but can be more difficult to interpret. The sum of all Sobol’ indices is equal to 1.

**Fig 5:**
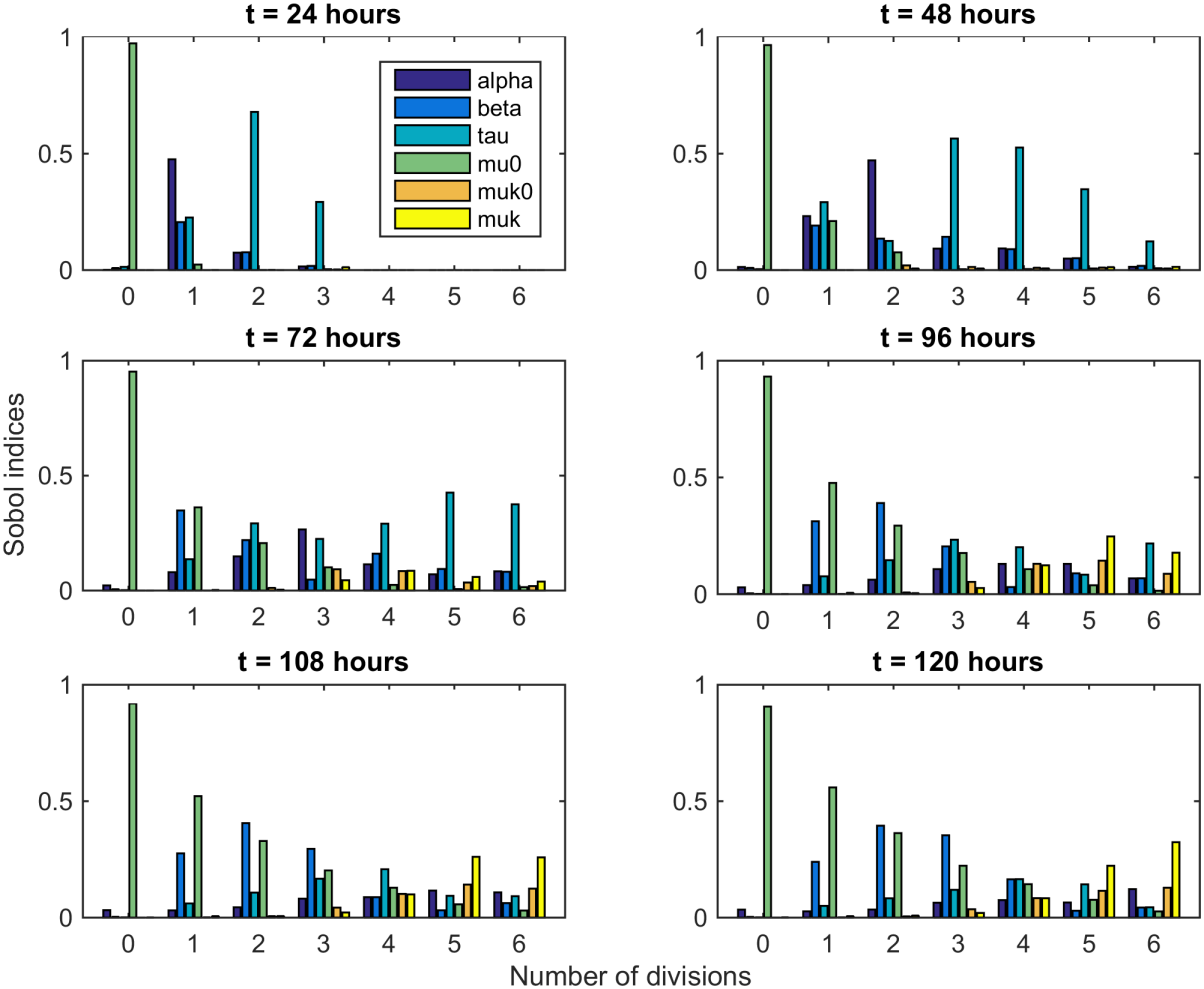
Sensitivity analysis with first order Sobol’ indices for each model parameter. Sobol’ indices are given for each model parameter, in each generation and for 6 given times. Dark blue bar represents the impact of the rate of non-divided cells into the resting phase *α*. Blue bar represents the impact of *β*, standing for the rate of entry into active phase. Turquoise bar represents the impact of the duration of the active phase *τ*. Green bar represents the impact of non-divided cell death rate *μ*_0_. Finally, orange and yellow bars represent the impact of cell death rate.

In this work, we only present first order Sobol’ indices (Eq (36)). They allow us to quantify the impact of a model parameter on the total number of cells in a given generation and for a given time. Each sub-figure in Fig 5 displays Sobol’ indices for each parameter (different colors) in each generation (*x*-axis). The six sub-figures represent a different time (corresponding to observation times of experimental data presented in Section 4.1).

As expected, parameter *μ*_0_ has a huge impact on cell dynamics for the early generations. During the first 72 hours of the experiment, the parameter *τ* most impacts cell division. This seems reasonable as *τ* is the time a cell has to stay in the active phase before dividing. Therefore, the number of cells in each generation depends on the duration of the active phase. However, once the division process is started, we notice that the impact of the rate of entry into phase active *β* increases. This means that after a certain time, duration and variability of the resting phase have a larger effect on the dynamics than the duration of the active phase. We also note that the effect of the death rates appears in the late times and for the larger generations. This is due to the fact that we consider that only cells that have divided several times can die, as has been shown in Section 4. Besides, sums of first order Sobol’ indices for each generation and time are close to 1 for most of them. This means that most of the time, parameters do not interact with each other to impact the variability of the number of cells.

## 6 Discussion

The use of CFSE labelled cell data allows one to study cell proliferation. In this study, we focused on the division of immune cells (CD4+ and CD8+) to estimate key parameters of this process using mathematical modelling. We improved the model presented in [20] by adding a latency phase that allows us to describe the delay between stimulus and cell response and to represent the behaviour of non-divided cells. Although the hypothesis of a first division described by an exponential distribution may be questionable [32, 33], it allows us to derive explicit solutions from the equations, ensuring the existence, uniqueness and non-negativity of solutions. It also allows us to derive first estimates of each parameter that adequately describe experimental data for all observation times. Moreover, we are able to reproduce immune cell behaviour using a model with relatively few parameters to estimate (6 parameters) compared to the branching process model or the Cyton model (11 parameters each) presented in [12] and used to reproduce the same data as in our study. We furthermore show that our model performs better than the branching process models and the Cyton model [12] in terms of AICc when model parameters are estimated using data in log_10_ scale. However, we chose to estimate model parameters based on raw data instead of data in log_10_ scale. We think it is more appropriate to use a linear cell numbers when presented in a histogram (or density) format, when the objective is to estimate average key parameters of the division kinetics. This makes the fit more robust against possible subset of rapidly proliferating or quiescent cells. The log-scale is more appropriate for fitting time series with exponential kinetics.

Recent evidence suggests that duration of both G_1_ phase and S/G_2_/M phases is highly variable [34]. In our model, we assume that only the resting phase has a probabilistic duration. Although this phase is often thought to contain G_0_ and G_1_ phases, this distinction is not required and it can therefore includes a part of S/G_2_/M phases. In that case, phases S/G_2_/M can have a variable duration. To go further, one could assume that the parameter *τ* is randomly distributed, but CFSE data may not be rich enough to estimate the part of variability from resting and active phases.

Applying this model to experimental CFSE data allows us to compare CD4+ and CD8+ cell dynamics. Both type of cells are better described with the three-phase model, assuming a positive loss of non-divided cells, and death rates depending on the number of divisions for the other generations. Our model is simple enough to derive explicit solutions, and remains sufficiently accurate to fit experimental data with few parameters to estimate. We believe that this three-phase model gives valuable insights into immune cell response, in terms of dynamics and kinetic parameters. It may be used to analyse different cell responses and may help to identify pathological division processes.

We performed a sensitivity analysis to quantify the impact of each model parameter on cell division dynamics. Interestingly, we found that parameters do not have the same impact all the time and for different generations. While the duration of the active phase *τ* has a large impact for the early times of the experiment, it seems that the rate of entry into the active phase *β* has a larger effect on the dynamics during the late times. Although these results should be assessed *via* biological experiments, we believe that they could help biologists to better understand cell division mechanisms and kinetics.
